# Диагностическая значимость компьютерной томографии в комплексном обследовании больных с адренокортикальным раком

**DOI:** 10.14341/probl12846

**Published:** 2022-04-25

**Authors:** В. Ф. Русаков, И. Е. Щербаков, И. К. Чинчук, Т. В. Савельева, Д. В. Реброва, О. И. Логинова, Т. С. Придвижкина, Р. А. Черников, Л. М. Краснов, Ю. Н. Федотов, Е. А. Федоров, И. В. Саблин, И. В. Слепцов, Ш. Ш. Шихмагомедов, Е. А. Згода

**Affiliations:** Санкт-Петербургский Государственный университет, Клиника высоких медицинских технологий им. Н.И. Пирогова; Санкт-Петербургский Государственный университет, Клиника высоких медицинских технологий им. Н.И. Пирогова; Санкт-Петербургский Государственный университет, Клиника высоких медицинских технологий им. Н.И. Пирогова; Санкт-Петербургский Государственный университет, Клиника высоких медицинских технологий им. Н.И. Пирогова; Санкт-Петербургский Государственный университет, Клиника высоких медицинских технологий им. Н.И. Пирогова; Санкт-Петербургский Государственный университет, Клиника высоких медицинских технологий им. Н.И. Пирогова; Санкт-Петербургский Государственный университет, Клиника высоких медицинских технологий им. Н.И. Пирогова; Санкт-Петербургский Государственный университет, Клиника высоких медицинских технологий им. Н.И. Пирогова; Санкт-Петербургский Государственный университет, Клиника высоких медицинских технологий им. Н.И. Пирогова; Санкт-Петербургский Государственный университет, Клиника высоких медицинских технологий им. Н.И. Пирогова; Санкт-Петербургский Государственный университет, Клиника высоких медицинских технологий им. Н.И. Пирогова; Санкт-Петербургский Государственный университет, Клиника высоких медицинских технологий им. Н.И. Пирогова; Санкт-Петербургский Государственный университет, Клиника высоких медицинских технологий им. Н.И. Пирогова; Санкт-Петербургский Государственный университет, Клиника высоких медицинских технологий им. Н.И. Пирогова; Санкт-Петербургский Государственный университет, Клиника высоких медицинских технологий им. Н.И. Пирогова

**Keywords:** адренокортикальный рак, инциденталома надпочечника, новообразование надпочечника, компьютерная ­томография, стероидный профиль мочи, газовая хромато-масс-спектрометрия, стероидогенез, синдром Кушинга

## Abstract

**ОБОСНОВАНИЕ:**

ОБОСНОВАНИЕ. Большинство случаев выявления новообразований надпочечников происходит случайно, при проведении визуализирующих исследований по поводу других заболеваний. Подобные находки трактуются как инциденталомы надпочечников. Общепопуляционная статистика их выявления при выполнении КТ-исследований приближается к 4%. В структуре всех новообразований надпочечников адренокортикальный рак (АКР), по данным разных авторов, составляет 4–12%. Из всех методов визуализации надпочечников в настоящее время наиболее диагностически значимым общепринято считать КТ-исследование с болюсным введением контрастного вещества и стандартным расчетом рентгеновской денситометрической плотности опухоли. Проведен анализ результатов КТ-визуализации у 67 больных АКР по единому протоколу. Описаны основные признаки, характерные для этого заболевания. Распознавание типичных для АКР критериев, выявляемых при КТ, имеет исключительное значение для дооперационной верификации высокого риска АКР, так как обоснованное подозрение на возможную злокачественность новообразования надпочечника при предоперационном обследовании крайне важно для выбора тактики хирургического лечения.

**ЦЕЛЬ:**

ЦЕЛЬ. Оценить значимость КТ как основного метода дооперационной диагностики у пациентов со злокачественными опухолями коркового слоя надпочечника. Изучить КТ-семиотику АКР на большой группе пациентов при исследовании по единому стандартному протоколу визуализации. Найти основные рентгенологические симптомы, характерные для АКР.

**МАТЕРИАЛЫ И МЕТОДЫ:**

МАТЕРИАЛЫ И МЕТОДЫ. Представлены результаты ретроспективного исследования данных КТ 67 пациентов с АКР, проходивших лечение в отделении эндокринной хирургии Клиники высоких медицинских технологий им. Н.И. Пирогова Санкт-Петербургского государственного университета (КВМТ СПбГУ) в период 2012–2020 гг. Проведена оценка диагностической значимости КТ у больных АКР.

**РЕЗУЛЬТАТЫ:**

РЕЗУЛЬТАТЫ. Для АКР наиболее характерны: неоднородная структура опухоли (84,3%), исследований, размеры опухоли от 3 до 9 см (75%), признаки инвазии в окружающие анатомические структуры (10%), денситометрическая нативная плотность опухоли выше +30 HU (75%), показатель абсолютного контрастного вымывания менее 60% (68,6%), показатель относительного контрастного вымывания менее 40% (64,6%).

**ЗАКЛЮЧЕНИЕ:**

ЗАКЛЮЧЕНИЕ. Ни одного достоверного фенотипического патогномоничного признака АКР при КТ с внутривенным контрастированием выявить не удалось. Вместе с тем КТ по стандартному протоколу должна быть проведена всем больным с подозрением или выявленным другим способом визуализации новообразованием надпочечников. Контрастное исследование с болюсным введением должно проводиться у всех пациентов, в случае наличия денситометрической плотности выше +5 Ед HU при оценке нативных изображений.

## Abstract (en)

**BACKGROUND:**

BACKGROUND: In most cases adrenal tumours are detected by accident while performing medical imaging tests for other diseases. These findings are treated as adrenal incidentaloma. Prevalence of incidentalomas detected on CT scans is up to 4%. According to different authors, 4–12% of all adrenal tumours are adrenocortical carcinomas. As for today, the most significant medical imaging technique is CT scan with bolus IV injection of contrast agent and assessment of tumour’s density. The analysis of the results of CT imaging in 67 patients with ACC was carried out according to a single protocol. The main signs characteristic of this disease are described. It is very important to evaluate typical signs of ACC on CT scans for risk assessment of ACC before surgical treatment. If malignant tumour is suspected during preoperative examination, it is extremely important to choose the right surgical treatment strategy.

**AIM:**

AIM: To evaluate the significance of CT as the main method of preoperative diagnosis in patients with malignant tumors of the adrenal cortex. Studying CT semiotics of adrenocortical cancer in a large group of patients using a single standard imaging protocol. Find the main radiological symptoms characteristic of adrenocortical cancer

**MATERIALS AND METHODS:**

MATERIALS AND METHODS: Here are the results of retrospective study of CT scans performed on 67 patients with adrenocortical carcinoma who received treatment in the Department of Endocrine Surgery of Saint-Petersburg State University N.I. Pirogov Clinic of High Medical Technologies during 2012–2020. The diagnostic significance of CT in patients with ACC was assessed.

**RESULTS:**

RESULTS: The most common features of ACC: tumour heterogeneity (84.3%), tumour’s size 3–9 cm (75%), signs of invasion into surrounding structures (10%), pre-contrast density above +30 HU (75%), absolute contrast washout less than 60% (68.8%), relative contrast washout less than 40% (64.6%)

**CONCLUSION:**

CONCLUSION: CT scan with IV contrast was not able to show any definitive pathognomonic signs of ACC. Nevertheless, CT scan should be performed in all patients with suspected (or confirmed using other medical imaging technique) adrenal tumour according to standard protocol. Bolus injection of contrast agent should be performed in all patients with tumour’s pre-contrast density above +5 HU.

## ОБОСНОВАНИЕ

Со времени первого анатомического описания надпочечников в XVI в. Бартоломео Эустахио или Евстахием (Eustachio Bartolomeo 1510–1574) [1] и новообразования надпочечника (F. Fränkel, 1886) [2] представления о распространенности иморфологической структуре этих опухолей к настоящему времени значительно расширились. Накопление знаний и технологический прогресс в медицине привели к «взрывному», экспоненциальному росту количества выявляемых опухолей надпочечников в последние десятилетия. Основная причина этого роста числа диагностируемых опухолей надпочечников — внедрение в клиническую практику современных визуализирующих методов диагностики: ультразвукового исследования (УЗИ), компьютерной томографии (КТ), магнитно-резонансной томографии (МРТ) [3–7]. Оказалось, что новообразования надпочечников не относятся к редкой патологии. Общепопуляционная статистика их выявления при выполнении КТ исследований приближается к 4%. Заболеваемость существенно увеличивается с возрастом [8–10]. Абсолютное большинство случаев выявления новообразований надпочечников происходит случайно, при проведении визуализирующих исследований по поводу других заболеваний (например, при УЗИ органов брюшной полости или при КТ органов грудной клетки). Подобные находки трактуются как инциденталомы надпочечников (от англ. incidental discovery of an adrenal mass — случайно выявленное новообразование надпочечника) [11]. При первичном выявлении инциденталомы надпочечника для выбора тактики дальнейшего ведения больного и, самое главное, прогноза, решающее значение имеют несколько факторов [7][12–18].

Наличие высокого онкологического риска, гормональной гиперпродукции определенной степени, инвазивный рост или сдавление соседних органов, предполагает в абсолютном большинстве случаев хирургическое лечение. В тоже время при отсутствии этих проявлений, возможно динамическое наблюдение либо медикаментозная коррекция гормональных нарушений.

## Морфология и эмбриология опухолей

Морфологическая структура новообразований надпочечников чрезвычайно разнообразна. Спектр морфологической структуры всех новообразований надпочечников очень широк — они разделяются на следующие большие группы (18, 24).

Из всех методов визуализации надпочечников в настоящее время наиболее диагностически значимым, общепринято считать КТ исследование с болюсным введением контрастного вещества и стандартным расчётом рентгеновской денситометрической плотности опухоли в фиксированных по времени точках [18][19]. Вместе с тем, в клинической практике существует еще один современный метод визуализации надпочечников — МРТ. МРТ на сегодняшний день является более дорогим, менее доступным в практической работе, более продолжительным по времени исследованием (по сравнению с КТ) и, самое главное, по мнению некоторых авторов, при выполнении МРТ невозможно достоверно оценить предполагаемую морфологическую структуру опухоли и, соответственно, ее злокачественный потенциал [17][20][21]. Расширение технологических и финансовых возможностей привело к более широкому использованию методов ядерной медицины, прежде всего, позитронно-эмиссионной томографии с различными радиофармпрепаратами, но доступность ее остается недостаточной и применяется она только по ограниченным показаниям, как правило, после проведения КТ или МРТ [12][22].

Что касается значительно более дешевого и общедоступного ультразвукового исследования надпочечников, то для проведения исследований надпочечников оно категорически не рекомендуется в связи с низкой чувствительностью [4][20][23], и может использоваться после КТ или МРТ для подтверждения диагноза киста или липома [24].

Из множества возможных вариантов опухолевых поражений надпочечника в плане своевременности и максимальной точности дооперационной диагностики на выживаемость больного безусловно влияет раннее выявление адренокортикальной карциномы (син.: рак коркового слоя надпочечника, адренокортикальный рак (АКР)) [25–27].

АКР — редкая агрессивная опухоль, исходящая из коркового вещества надпочечников, которая чаще диагностируется поздно, с большой опухолевой массой, метастатическими поражениями регионарных лимфоузлов и отдаленными гематогенными метастазами. Распознавание типичных для АКР клинических, биохимических параметров и данных визуализации необходимо для своевременной диагностики, определения объема оперативного вмешательства и раннего применения соответствующей терапии [12].

В структуре всех новообразований надпочечников АКР, по данным разных авторов, составляет 4–12% [4][28–30]. Казалось бы, при современном развитии визуализации и технических возможностей взятия биопсии под непосредственным визуальным контролем, а также применения морфологических исследований с использованием современных окрасок и иммуногистохимических методов, проблемы установления точного морфологического диагноза до проведения оперативного вмешательства должны быть решены. Однако, это далеко не так, и при планировании биопсии возникают сложности, зачастую непреодолимые, — прежде всего связанные с анатомическими особенностями расположения опухоли. При этом большие опухоли могут значительно изменять топографию анатомической области. По мнению Soyer P et al. (1998), при проведении пункционной биопсии возможно повреждение капсулы новообразования и распространение ткани опухоли по ходу иглы [31]. П.С. Ветшев и соавт. (2005) и R. Kuruba et al. (2008) отмечают, что при проведении биопсии достаточно высок риск кровотечения и развития жизнеугрожающего осложнения — симпатоадреналового криза с синдромом неуправляемой гемодинамики при феохромоцитоме [32][33]. Также весьма часто встречаются серьезные трудности в интерпретации при оценке полученного морфологического материала.

Именно в связи с этим, исключительное значение для дооперационной верификации высокого риска АКР имеют неинвазивные визуализирующие методы исследования (КТ, МРТ), так как обоснованное подозрение на возможную злокачественность новообразования надпочечника при предоперационном обследовании крайне важно для выбора тактики хирургического лечения [34–36].

Публикации, посвященные КТ-семиотике АКР, немногочисленны (табл. 1), а анализируемые в них случаи, как правило, единичны [3][5][12][20][22][29][35][37].

**Table:** Таблица 1. Публикации, посвященные лучевой диагностике АКРTable 1. Publications on radiodiagnosis of adrenocortical cancer

Автор	Год публикации исследования	Число наблюдений АКР
М. Eghrari, et al.	1980	3
S. Hussain, et al.	1985	7
Т.Н. Трофимова и соавт.	2004	14
J.M.A. Slattery, et al.	2006	7
М.Е. Белошицкий и соавт.	2007	25
Т.В. Солдатова	2011	12
S. Petersenn, et al.	2015	51
Н.А. Майстренко и соавт.	2016	16
Шингареева Л.А. и соавт.	2018	3
N. Yilmaz, et al.	2020	11

Так, число случаев АКР, подвергнутых анализу в этих работах, в среднем составляет 10–15 наблюдений. Исключение составляет работа S. Petersenn et al. (2015) — 51 случай АКР, в которой, правда, изучались клинические наблюдения из разных медицинских центров с различными протоколами визуализации [38].

По мнению многих авторов, такие признаки как большие размеры опухоли, многоузловое строение, неоднородность структуры, нечеткие контуры, наличие кальцинатов, инфильтративный рост и наличие метастазов, могут уверенно свидетельствовать о злокачественности опухоли надпочечника [3][12][13][36][39]. Вместе с тем при некоторых АКР многие из указанных признаков при КТ или МРТ не выявляются [13][20][40–42].

Преобладающая часть авторов указывают на высокий онкологический потенциал опухолей надпочечников размером более 4 см [12][15][43][44]. Однако встречаются публикации, где в исследованиях выявлены адренокортикальные злокачественные опухоли размерами исключительно более 5–6 см. Авторы полагают, что именно такой размер является критерием малигнизации опухоли надпочечника при проведении КТ исследования [20][14][45]. Необходимо учитывать, что именно размер в сочетании с гистологическими особенностями опухоли при АКР является основным фактором неблагоприятного прогноза. Вместе с тем встречаются работы, где исследователи выявили весьма небольшие АКР размерами 3,25 см [40], 2,8 см [13] и даже 2 см [38][46]. В частности, А.В. Араблинский и соавт. (2011) высказывают следующее соображение относительно связи размеров опухоли и ее возможной злокачественности: объемное образование диаметром более 3 см в 90–95% случаев является злокачественным, а менее 3 см в 78–87% — доброкачественным [47]. По данным большинства авторов, при КТ-исследовании АКР представляет собой образование с неровными нечеткими контурами [12][20][22][36][48][49]. Форма образования может определяться, как овальная, так и неправильная или многоузловая [22][23][44][50]. Важным индикатором злокачественности опухоли коркового слоя надпочечников является наличие неоднородности (гетерогенности) опухолевой массы, выявляемой при КТ-исследовании [44][49][51], особенно в сочетании с большими размерами новообразования [15]. Так, по данным Т.Н. Трофимовой и соавт. и E.K. Fishman et al., гетерогенность опухоли у больных АКР была выявлена в 100% наблюдений [23][51], тогда как в аденомах гетерогенность выявляется в 50% наблюдений [38]. Гетерогенность, как правило, связана с наличием тех или иных «включений» в опухолевую ткань, определяющихся на КТ. Речь идет о кальцификатах, некрозах, кистах и кровоизлияниях. Именно такие признаки некоторые исследователи отмечают, как дополнительный критерий АКР [12][14][15][22][44]. Кальцификаты, как микро (до 3 мм), так и макро (более 3 мм) встречаются в 28–30% наблюдений [7][12][39][45][52]. Как правило, они располагаются в центре раковой опухоли. Е.К. Fishman et al. (1987) отмечают, что некроз опухоли неизменно присутствовал в анализируемой им группе наблюдений в случае размеров злокачественной опухоли более 6 см [51]. Одновременно N. Schieda et al. (2017) опубликовали сообщение о крупных доброкачественных аденомах надпочечника (более 5 см в диаметре), у которых также выявлялись некрозы, кальцинаты и кровоизлияния, что имитировало КТ картину злокачественности процесса [41]. По мнению некоторых авторов (Fishman Е.К. et al., 1987), индикатором АКР является наличие тонкой опухолевой капсулы, выявляемой при рентгеноконтрастном исследовании в 30–50% случаев [51], а по некоторым данным — в 81% [22]. По данным же других исследователей, капсула у злокачественных адренокортикальных опухолей встречается редко [20]. Немаловажным критерием для диагностики АКР является инфильтративный рост в окружающие органы и сосудистые структуры (прежде всего, в печень и поджелудочную железу) и наличие метастазов в регионарные лимфатические узлы, что может указывать на злокачественность опухоли надпочечника [7][22]. По данным N. Bharwani et al., 2011, вовлечение почечной вены (до 20% наблюдений) чаще встречается при опухоли правого надпочечника и проявляется распространением опухоли в просвете этой вены [5]. Многие авторы в своих наблюдениях отметили, что характерный симптом АКР — это поражение нижней полой вены, проявляющийся в виде опухолевого тромба, и он выявляется в 15% наблюдений [44][53][54]. Н.В. Молашенко и соавт. (2010) отмечают, что чаще такая картина диагностируется при правосторонней локализации АКР (в80% наблюдений) [49]. Вместе с тем, встречаются публикации, где указано, что инвазивным ростом обладает не только АКР, но и другие новообразования надпочечника. Так, С. Cuevas et al., 2006 [42] выявили, что поражение нижней полой вены было связано с лейомиосаркомой надпочечников.

Важным диагностическим критерием для верификации АКР является нативная денситометрическая плотность и характеристики вымывания контрастного препарата из ткани надпочечника. Для оценки последних специалисты лучевой диагностики [8][52][55] используют показатели абсолютного и относительного вымывания контрастного препарата, рассчитанные по следующим формулам:

Абсолютное вымывание=(HU венозная фаза) - (HU отсроченное) / (HU венозная фаза) - (HU нативная фаза)] x 100%;

Относительное вымывание контраста=(HU венозная фаза) - (HU отсроченное) / (HU венозная фаза) x 100%;

где HU — значение денситометрической плотности в единицах Хаунсфильда.

Совокупные данные, полученные для идентификации аденом надпочечников, показывают, что AКР редко имеют значение нативной денситометрической плотности менее +10 HU [12]. Одновременно характерными показателями нативной денситометрической плотности для АКР многие исследователи считают диапазон от +25 HU до +45 HU [7][13][20][22]. Точно так же АКР сохраняют внутривенный контрастный материал и имеют абсолютный и относительный процент вымывания менее 60% и менее 40%, соответственно, через 15 минут после введения контраста [15][56][57] или менее 50% и менее 40%, соответственно, через 10 минут [52][57][58]. Вместе с тем M.J. Sangwaiya et al. [55] указывают на низкую чувствительность методики, где оценка контрастирования проводится через 10 минут, отмечая ее низкую чувствительность (76,8%) и специфичность (93,7%). По мнению же T. Pamela et al. (2009), большой размер и неоднородность являются более надежными показателями диагноза, чем значения вымывания [15].

В целом при анализе литературы, посвященной лучевой семиотике АКР, обращает на себя внимание тот факт, что публикации на эту тему редки, порой противоречивы, а анализируемые в них показатели нестандартны по протоколу визуализации.

## ЦЕЛЬ ИССЛЕДОВАНИЯ

## Цели исследования

## МАТЕРИАЛЫ И МЕТОДЫ

## Место и время проведения исследования

Место проведения. Отделение эндокринной хирургии Клиники высоких медицинских технологий им. Н.И. Пирогова Санкт-Петербургского государственного университета (КВМТ СПбГУ).

Время исследования. Исследование проводилось в период с 2012 по 2020 г.

## Изучаемые популяции (одна или несколько)

Основная группа — больные АКР.

Контрольная группа — больные с аденомами коркового слоя надпочечника.

Критерии включения в основную группу: все пациенты с АКР вне зависимости от пола, старше 18 лет.

Критерии исключения из основной группы: рецидив АКР, метастаз в надпочечник раковых опухолей иных локализаций, феохромоцитома.

Критерии включения в контрольную группу: все пациенты с аденомами в независимости от пола, старше 18 лет.

## Способ формирования выборки из изучаемой популяции

Сплошной.

## Дизайн исследования

Ретроспективное одноцентровое наблюдательное исследование.

## Описание медицинского вмешательства (для интервенционных исследований)

Исследование выполнялась на компьютерных томографах Aquilion 64 Toshiba с 64 рядами детекторов и Aquilion One Canon c 320 рядами детекторов. Всем обследованным пациентам после нативного сканирования внутривенно болюсно вводили 100 мл контрастного препарата «Омнипак» или «Ультравист» с концентрацией йода 350 мг/мл со скоростью 3,5–4 мл/с с помощью автоматических болюсных инжекторов производителя «Ulrich medical». Постконтрастное сканирование включало артериальную фазу (на 29–30 с после начала введения контрастного препарата), в венозную фазу (на 60–70 с) и в позднюю отсроченную фазу с задержкой сканирования 10 мин. В основной группе в 6 наблюдениях по различным причинам внутривенного контрастирования при КТ не проводилось.

## Методы

Способом определения критерия включения в основную группу являлась гистологическая верификация диагноза с обязательной оценкой злокачественного потенциала по критериям L.M. Weiss (1989) и обязательным иммуногистохимическим исследованием с расчетом индекса пролиферативной активности КИ-67 [59]. Диагноз АКР устанавливался при наличии более 5 баллов при световой микроскопии и иммуногистохимических признаках злокачественного роста. В контрольную группу пациенты включались при наличии гистологического заключения — аденома надпочечника.

Оценены КТ характеристики новообразований коркового слоя надпочечника: размер, топография, форма, контуры, края и капсула опухоли, наличие включений, нативная денситометрическая плотность, коэффициент вымывания контраста, признаки инвазии опухоли. Показатели абсолютного и относительного вымывания контрастного препарата оценивали по стандартным формулам. Изучен гормональный статус пациентов с АКР (оценены концентрации альдостерона, ренина, альдостерон-ренинового соотношения, кортизола на фоне ночной пробы с 1 мг дексаметазона, свободный метанефрин и нормметанефрин, адренокортикотропного гормона, дегидроэпиандростерона).

## Статистический анализ

КТ характеристики опухолей надпочечников и показатели гормонального статуса больных фиксировались в электронной базе данных. При анализе данных использована статистическая программа Microsoft Exсel 2013 (компания Microsoft, США). Описательная статистика для количественных данных представлена в виде среднего и стандартного отклонения M (SD) в случае нормально распределенных выборок и в виде медианы и интерквартильного размаха Ме (Q1–Q3) для выборочных распределений, не согласованных с нормальным. Нормальность проверялась при помощи критерия Шапиро–Уилка. Статистическая значимость различий в размерах и нативной плотности опухолей надпочечников между группами обосновывалась сиспользованием критерия Манна-Уитни. Для всех случаев проверки гипотез в качестве порогового использовался уровень значимости 0,05. Статистические расчеты проводились в программе R версии 4.1.2.

## Этическая экспертиза

Учитывая ретроспективный дизайн научной работы, Комитет по биомедицинской этике постановил: при условии публикации данных в деперсонифицированном виде, исследование в этической экспертизе не нуждается (номер протокола 11/21 дата подписания 30.11.2021).

## РЕЗУЛЬТАТЫ

Изучены данные КТ 67 пациентов (основная группа) с АКР (из них у 3 выявлены опухоли обоих надпочечников). Распределение в основной группе по полу: мужчин — 16 и женщин — 51. Средний возраст составил 48,1 (14,3) года. Хирургическое вмешательство осуществлялось всем больным. В 31,3% случаях (21 наблюдение) у больных отмечался лабораторный гиперкортицизм, представленный у 9 пациентов (13,4%) возможной функциональной автономной гиперпродукцией кортизола (уровень кортизола от 51–140 нмоль/л при пробе с 1 мг дексаметазона) и лишь у 12 больных (17,9%) — клинико-лабораторными проявлениями синдрома Кушинга. Для сравнения проведена оценка результатов в контрольной группе пациентов (n=564) саденомами коркового слоя надпочечников.

Размер опухолей в группе больных с первично выявленным АКР составлял 7,05 (5,85–7,67) см при минимальном значении 3 и максимальном — 23 см. Данные о размерах новообразований представлены на рис. 1. Злокачественные адренокортикальные опухоли мы условно разделили в зависимости от размера на маленькие до 4 см (6 случ.), средние от 4 до 6 см (11 случаев), большие от 6 до 10 (41 случай) и гигантские (рис. 2) — более 10 см (12 случай). Следует заметить, что от размеров опухоли зависит выбор хирургического подхода: при опухолях небольшого размера без инвазии или лимфаденопатии выполнялась ретроперитонеоскопическая адреналэктомии, при наличии метастазов в регионарные лимфоузлы, инвазии ворганы и сосуды или крупной опухоли — открытая адреналэктомия. Особое внимание обращает на себя категория пациентов (8,6% наблюдений), имеющих малые размеры образования (от 3 до 4 см). Их, в силу небольших размеров, в клинической практике обычно не рассматривают с позиции онкологической настороженности, а именно в этой группе существует наибольшая вероятность несвоевременной диагностики злокачественной опухоли (рис. 3, 4).

**Figure:**
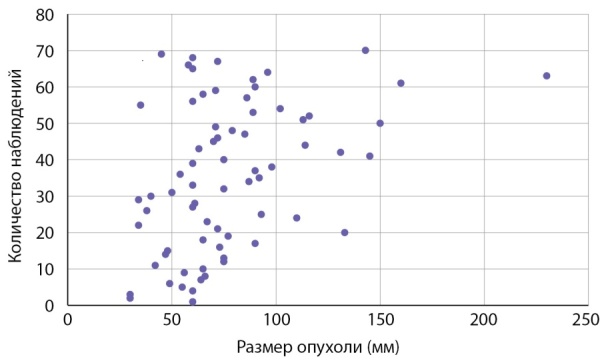
Рисунок 1. Распределение пациентов с адренокортикальным раком в зависимости от размеров опухоли.Figure 1. Distribution of patients with adrenocortical cancer depending on tumor size.

**Figure:**
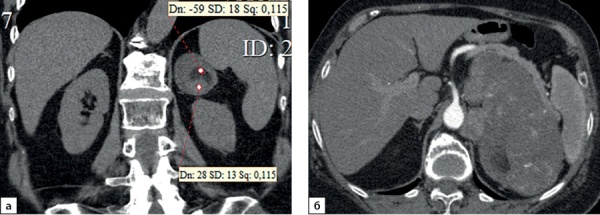
Рисунок 2. Компьютерные томограммы пациентки В., 70 лет с гигантской опухолью левого надпочечника (137х80 мм), деформирующая тело и хвост поджелудочной железы: а — изображение реформатированное в корональной плоскости, нативная фаза сканирования, в структуре мягкотканного образования с денситометрическими показателями до 28HU включения с низкой плотностью (до -59HU), соответствующие жировой ткани; б — артериальная фаза, включения жира не накапливают контрастный препарат.Figure 2. Computed tomography of patient V., 70 years old, with a giant tumor of the left adrenal gland (137x80 mm), deforming the body and tail of the pancreas: a — image reformatted in the coronal plane, native scanning phase, in the structure of the soft tissue formation with densitometric parameters up to 28HU inclusion with low density (up to -59HU), corresponding to adipose tissue; b — arterial phase, inclusions of fat do not accumulate a contrast agent.

**Figure:**
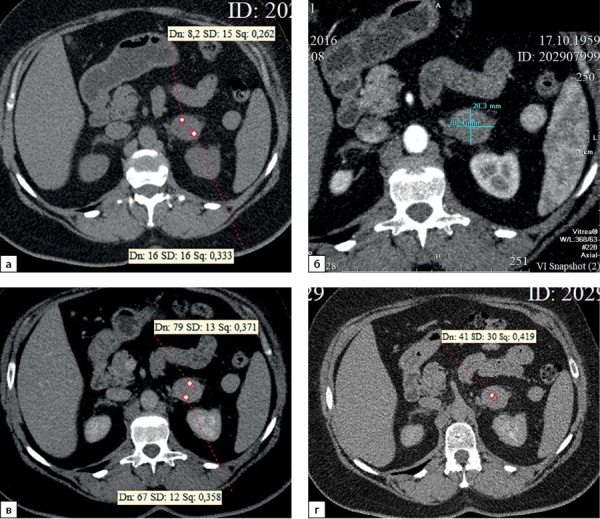
Рисунок 3. Компьютерные томограммы пациентки И., 67 лет с малыми размерами адренокортикальной карциномы (28х35 мм), распространяющейся из латеральной ножки левого надпочечника вдоль передней поверхности почки: а — изображение в аксиальной плоскости, нативная фаза сканирования — показатели плотности до 16 HU; б — артериальная фаза постконтрастного сканирования; в — венозная фаза; г — фаза отсроченного сканирования, коэффициент абсолютного вымывания контрастного препарата составил 42%, относительного — 31,8%.Figure 3. Computed tomography of patient I., 67 years old, with small size adrenocortical carcinoma (28x35 mm), spreading from the lateral pedicle of the left adrenal gland along the anterior surface of the kidney: a — image in the axial plane, native scanning phase — density values up to 16 HU; b — arterial phase of post-contrast scanning; c — venous phase; d — phase of delayed scanning, the coefficient of absolute washout of the contrast agent was 42%, relative — 31.8%.

**Figure:**
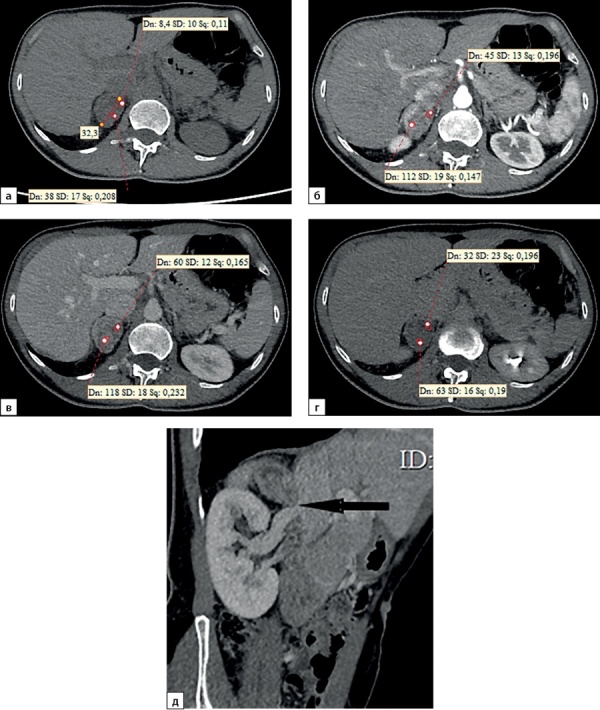
Рисунок 4. Компьютерные томограммы пациентки Ч., 42 лет с небольшой адренокортикальной карциномой (32х28 мм), с гетерогенной опухолевой тканью, распространяющейся до ворот почки: а — изображение в аксиальной плоскости, нативная фаза сканирования, плотность от +8,4 до +38HU; б — артериальная фаза постконтрастного сканирования; в — венозная фаза; г — фаза отсроченного сканирования, д — изображение реформатированное в косой сагиттальной плоскости, венозная фаза сканирования, образование локально деформирует почечную вену (стрелка). Коэффициент абсолютного вымывания контрастного препарата составил 56%, относительного — 36%.Figure 4. Computed tomography of patient Ch., 42 years old, with a small adrenocortical carcinoma (32x28 mm), with heterogeneous tumor tissue extending to the hilum of the kidney: a — image in the axial plane, native scanning phase, density from +8.4 to +38HU ; b — arterial phase of post-contrast scanning; c — venous phase; d — phase of delayed scanning, e — image reformatted in the oblique sagittal plane, venous phase of scanning, the mass locally deforms the renal vein (arrow). The coefficient of absolute washout of the contrast agent was 56%, relative — 36%.

Как следует из данных приведенных на рисунке 1 и табл. 2, первичная диагностика АКР происходит при больших размерах опухолей. Размеры опухолей более 7 см были выявлены в половине наблюдений, в четверти наблюдений (25% случаев) опухоли были более 9 см. 50% адренокортикальных раковых опухолей имеют размеры от 59,5 до 90 мм.

**Table:** Таблица 2. Данные о размерах АКРTable 2. Data on the size of adrenocortical cancer

Минимальный размер опухоли	30 мм
25% больных	до 59,5 мм
50% больных	до 70 мм
25% больных	более 90 мм
Максимальный размер опухоли	230 мм

Мы провели оценку размеров доброкачественных аденом коркового слоя надпочечников в контрольной группе, для сравнения с размерами опухолей у пациентов с АКР. Размер аденом составлял 35,0 (26,0–35,7) мм и варьировал от 8 до 97 мм (табл. 3). Сравнение с размерами АКР в основной группе, составившими 70,5 (58,5–76,7) мм, показало, что адренокортикальные раковые опухоли имеют статистически значимо большие размеры, чем аденомы, р<0,001. У больных с аденомами надпочечников 50% образований не превышают 35 мм и в четверти случаев были более 44 мм (табл. 3). Обращает на себя внимание, что большая часть аденом (75%) имели размеры от 8 мм до 5 см (рис. 5, 6 и табл. 3), тогда как у пациентов с АКР большая часть опухолей (75%) имели размеры от 3 до 9 см (рис. 1, табл. 2). Вместе с тем, при изучении размеров опухолей у больных с новообразованиями надпочечников (аденомами и АКР) выявлено, что лишь в 14,7% случаях при опухолях более 4 см выявлялся рак коры надпочечника. С другой стороны, среди больных с аденомами надпочечника опухоли более 6 см встречаются лишь в 5% наблюдений (рис. 5, 6), тогда как более 75% АКР имеют размер более 6 см (табл. 2).

**Table:** Таблица 3. Данные о размерах аденомы надпочечниковTable 3. Data on the size of adrenal adenoma

Минимальный размер опухоли	8 мм
25% больных	до 26 мм
50% больных	до 35 мм
25% больных	более 44 мм
Максимальный размер опухоли	97 мм

**Figure:**
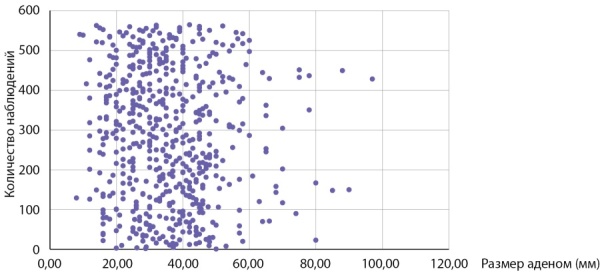
Рисунок 5. Распределение пациентов с аденомами надпочечников в зависимости от размеров опухоли.Figure 5. Distribution of patients with adrenal adenomas depending on tumor size.

**Figure:**
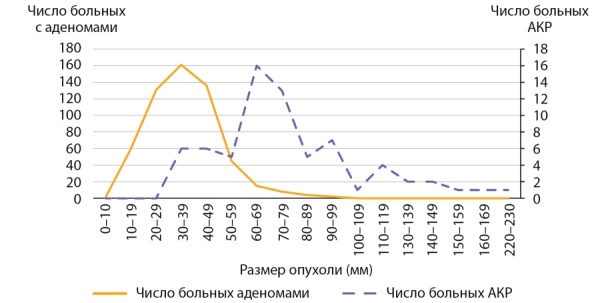
Рисунок 6. Распределение пациентов с аденомами надпочечников и АКР по группам в зависимости от размеров опухоли.Figure 6. Distribution of patients with adrenal adenomas and AKP by groups depending on tumor size

Неизмененная ткань надпочечника, наряду с опухолевой тканью, визуализировалась в половине наблюдений (52,9% набл.) АКР, в 48,1% раковых новообразований надпочечника неизмененная ткань при КТ не дифференцировалась. Ткань надпочечника выявлялась лишь в опухолях размерами, не превышающими 12 см. При АКР большего размера неизмененная ткань надпочечника при КТ не определяется вовсе.

Раковая опухоль относительно неизмененной ткани надпочечника могла располагаться в любом положении: медиально, ниже, латерально, выше или их сочетания (рис. 7). В анализируемой группе пациентов наиболее часто опухоль располагалась ниже (11 наблюдений), латерально (7 набл.) и выше (6 случ.) ткани надпочечника. Остальные локации встречались примерно в одинаковом числе наблюдений.

**Figure:**
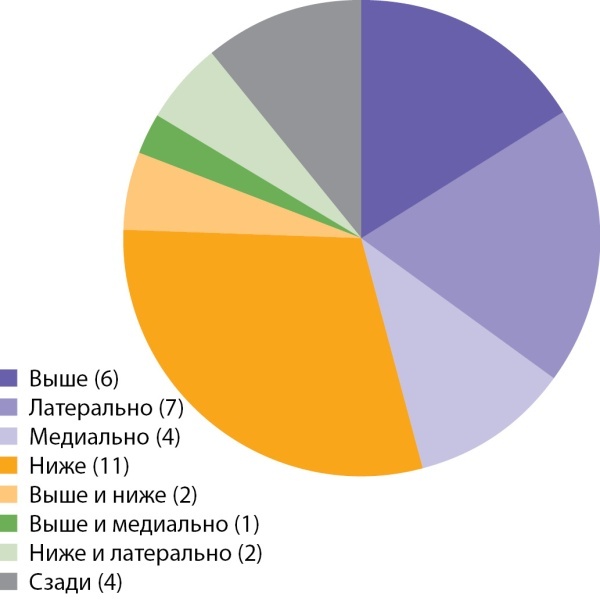
Рисунок 7. Распределение случаев АКР в зависимости от расположения раковой опухоли относительно неизмененной ткани надпочечника.Figure 7. Distribution of cases of adrenocortical cancer depending on the location of the cancer relative to the unchanged tissue of the adrenal gland.

Анатомической частью надпочечника, из которой происходил рост раковой опухоли, являлись, как правило, ножки надпочечника (30 наблюдений — 42,9%): латеральная (14 случаев), медиальная (12) или их сочетание (4). Реже опухоль распространялась из тела надпочечника (8) или из тела и ножек (1). Дифференцировать при КТ ­анатомическую зону надпочечника, из которой исходит АКР, не представлялось возможным в половине наблюдений (44,2%).

В половине наблюдений (в 48,6% случаев), АКР относительно тела почки располагался сверху, в трети наблюдений (34,3%) — кпереди от почки, а в 14,3% случаев — сверху и кпереди. Остальные локализации были крайне редки. В трети наблюдений (34% — 24 наблюдения) опухоль локализовалась в воротах почки.

Форма выявленных АКР была овальной в половине наблюдений (35 случаев — 50%), округлой в 18 случаях (25,7%) или неправильной в 17 случаях (24,3%). Контуры адренокортикальных раковых опухолей определялись как четкие в 92,9% случаев (65). В 3/4 наблюдений (75% — 53 случая) края опухоли были ровные. Необходимо отметить, что даже при крупных размерах контуры опухолей надпочечников были преимущественно четкими и ровными (60 набл. — 85,7%). У 31 (44,3%) пациента, как правило с наименьшими размерами образований, удалось визуализировать неизменные по размерам и структуре отделы надпочечника. В 27 (38,6%) наблюдениях опухоли достигали ворот ипсилатеральной почки. В большинстве наблюдений (72,6%) уАКР выявлялась капсула.

Однородная структура опухолей визуализировалась лишь у 11 (15,7%) пациентов с АКР, в остальных наблюдениях (84,3%) имела гетерогенную структуру. Неоднородность структуры придавали кальцинаты (рис. 8): в 7 наблюдениях мелкие, до 3 мм, в 8 — более крупные (всего у 15–21,4%), а также кистозные включения правильной (12) и неправильной формы 9 (всего в 21 случае — 30%) и зоны некроза (в 26 наблюдениях — 37,1%). Зоны некроза дифференцировались как участки относительно пониженной денситометрической плотности с нечеткими контурами, с минимальным накоплением контрастного препарата.

**Figure:**
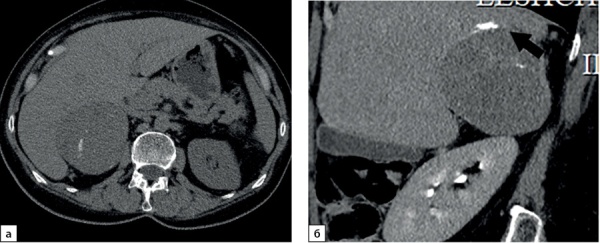
Рисунок 8. Компьютерные томограммы пациентки Л., 63 лет с опухолью правого надпочечника размерами до 72 мм, с полиморфными кальцинатами в структуре (стрелка): а — изображение в аксиальной плоскости, нативная фаза сканирования; б — фаза отсроченного сканирования, изображение реформатированное в сагиттальной плоскости.Figure 8. Computed tomography of patient L., 63 years old, with a tumor of the right adrenal gland up to 72 mm in size, with polymorphic calcifications in the structure (arrow): a — image in the axial plane, native scanning phase; b — delayed scan phase, image reformatted in the sagittal plane.

Медиана и интерквартильный размах максимальной нативной плотности АКР составили 32,0 (28,0–31,5) HU. Показатели нативной плотности в интервале от +10 до +30 HU отмечены у 22 больных (31,4%) пациентов, от +30 до +40 HU у 37 (52,9%) человек и свыше +40 HU в 10 (14,3%) наблюдений. Необходимо отметить, что в анализируемых наблюдениях встретился 1 пациент и с меньшей чем +10 HU нативной плотностью опухоли (рис. 9, 10). При ­выполнении денситометрии сознательно избегали попадания в зону измерения участков некроза, кистозных включений и кальцинатов.

**Figure:**
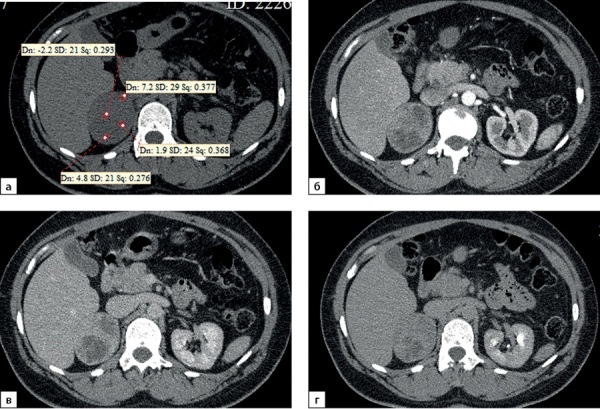
Рисунок 9. Компьютерные томограммы пациентки С., 44 лет с крупной опухолью правого надпочечника размерами (66 мм): а — изображение в аксиальной плоскости, нативная фаза сканирования; образование имеет четкие ровными контуры, умеренно диффузно неоднородную структуру с низкими денситометрическими показателями от +2,2 до +7,2 HU; б — артериальная фаза постконтрастного сканирования; в — венозная фаза; г — фаза отсроченного сканирования. Коэффициент абсолютного вымывания контрастного препарата составил 42%, относительного — 38,1%.Figure 9. Computed tomography of patient S., 44 years old, with a large tumor of the right adrenal gland (66 mm): a — image in the axial plane, native scanning phase; the formation has clear, even contours, a moderately diffusely heterogeneous structure with low densitometric values from +2.2 to +7.2 HU; b — arterial phase of post-contrast scanning; c — venous phase; d — delayed scan phase. The coefficient of absolute washout of the contrast agent was 42%, relative — 38.1%.

**Figure:**
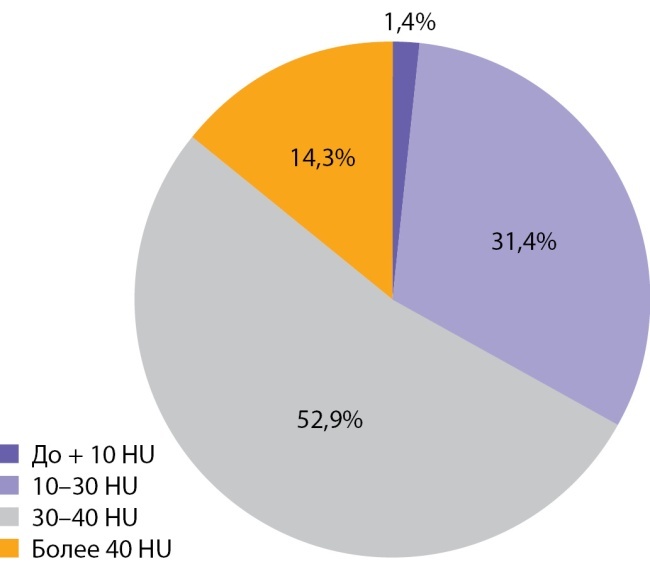
Рисунок 10. Распределение случаев АКР в зависимости от нативной плотности опухоли.Figure 10. Distribution of adrenocortical cancer cases depending on native tumor density

Для сравнения была проведена оценка нативной денситометрической плотности в группе пациентов с аденомами надпочечника (n=564). Медиана нативной денситометрической плотность выявленных аденом составила 10,0 ( -5,0–9,9) HU, что статистически значимо меньше плотности АКР, р <0,001. Сводные данные представлены в таблице 4 и на рисунке 11. 46,6% аденом надпочечников имели нативную денситометрическую плотность менее +10 HU. В остальных наблюдениях (53,4%) нативная плотность аденом превышала +10 HU, а в каждом десятом случае (13,3%) аденомы имели доконтрастную денситометрическую плотность +30 HU и больше.

**Table:** Таблица 4. Распределение пациентов с аденомами надпочечников в зависимости от показателей нативной плотностиTable 4. Distribution of patients with adrenal adenomas depending on native density indicators

Показатели плотности (HU)	от -36 до 0	от 0 до +10	от +10 до +20	от +20 до +30	от +30 до +50	Выше +50
Число наблюдений(n=564), %	173 (30,7)	90(15,9)	10(19,5)	109(19,5)	75(13,3)	7(0,01)

**Figure:**
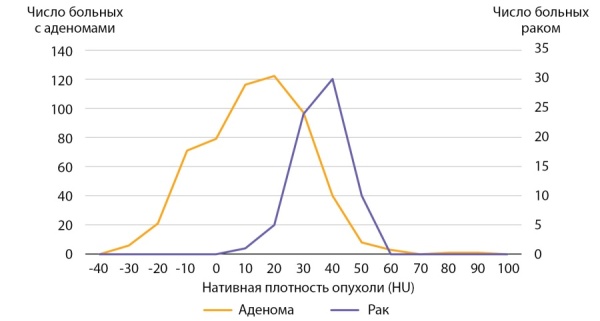
Рисунок 11. Распределение пациентов с аденомами надпочечников и АКР в зависимости от нативной плотности опухоли.Figure 11. Distribution of patients with adrenal adenomas and adrenocortical cancer depending on native tumor density.

Среди пациентов, которым выполнено исследование с внутривенным болюсным контрастированием (n=63), наиболее выраженное накопление контрастного препарата (оценивали по максимальному градиенту накопления и площади контрастированной ткани опухоли) наблюдали в подавляющем большинстве случаев в венозную фазу — у 53 (84,1%), в 6 (9,5%) наблюдениях — в артериальную фазу, и в 3 (4,7%) наблюдениях накопление контраста в артериальную и венозную фазу было сопоставимо по интенсивности. У 51 (80,9%) опухолей на фоне контрастирования отчетливо дифференцировалась «плотная» капсула по периферии.

Показатель абсолютного вымывания контрастного препарата при АКР составил от 10 до 87%, относительного — от 9 до 65% (рис. 12).

**Figure:**
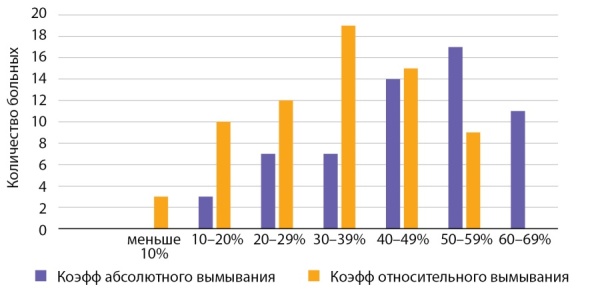
Рисунок 12. Распределение случаев АКР в зависимости от показателей коэффициента вымывания контрастного препарата.Figure 12. Distribution of cases of adrenocortical cancer depending on the washout coefficient of the contrast agent.

При размерах раковой опухоли до 40 мм показатель абсолютного вымывания от 10 до 87%, показатель относительного вымывания от 9 до 51%. При размерах АКР до 50 мм показатель абсолютного вымывания от 57 до 79%, показатель относительного вымывания контраста от 36 до 56%. При размерах опухоли свыше 10 см показатель абсолютного вымывания от 41 до 81%, показатель относительного вымывания контраста от 20 до 51%. Таким образом, выявлена обратная зависимость коэффициента вымывания контраста и размеров опухоли.

В 10% наблюдений (7 случаев) при КТ выявлены признаки инвазии опухоли в прилежащие анатомические структуры. Подобная ситуация встречается лишь у пациентов с опухолями большого размера. В 5 случаях при КТ определяется врастание опухоли в нижнюю полую вену (рис. 13), при этом в двух наблюдениях, ­кроме поражения вены (рис. 14), отмечена инвазия в ткань печени (1) и почки (1). В двух наблюдениях АКР распространялся на почку (1) и поджелудочную железу (1). В 8,6% наблюдениях (6 случаев) выявлялось увеличение забрюшинных лимфоузлов.

**Figure:**
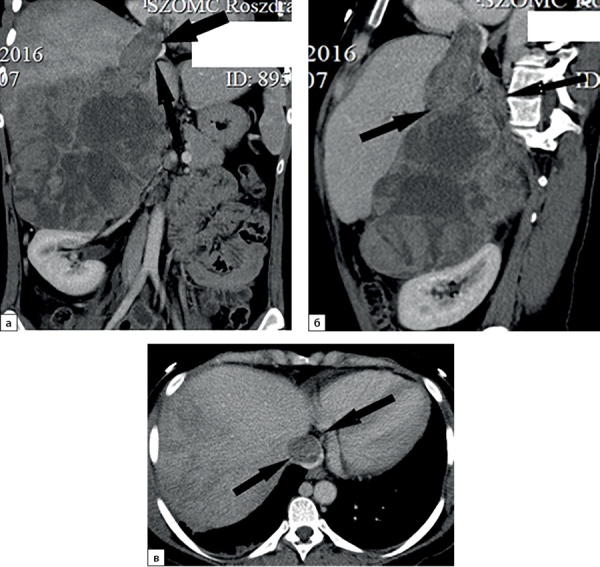
Рисунок 13. Компьютерные томограммы пациентки С., 24 лет, с гигантской опухолью правого надпочечника (165 мм), вызвавшей тромбоз нижней полой вены: а — изображение реформатированное в косой корональной плоскости, венозная фаза сканирования, опухолевый тромб в нижней полой вене (стрелки), пролабирующий в правое предсердие (стрелки); б — изображение реформатированное в косой сагиттальной плоскости, венозная фаза сканирования; в — венозная фаза, аксиальная плоскость.Figure 13. Computed tomography of patient S., 24 years old, with a giant tumor of the right adrenal gland (165 mm), which caused thrombosis of the inferior vena cava: a — image reformatted in an oblique coronal plane, venous phase of scanning, tumor thrombus in the inferior vena cava (arrows) , prolapsing into the right atrium (arrows); b — image reformatted in the oblique sagittal plane, venous phase of the scan; c — venous phase, axial plane.

**Figure:**
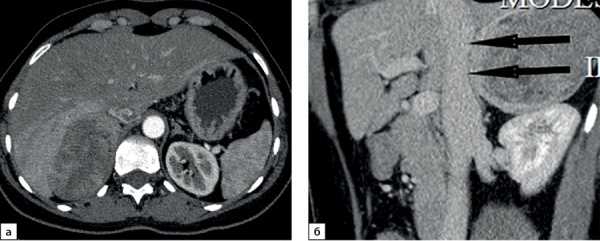
Рисунок 14. Компьютерные томограммы пациентки М., 41 года, с крупной опухолью правого надпочечника размерами до 90 мм: а — артериальная фаза постконтрастного сканирования, диффузно неравномерное накопление контрастного препарата, зона гиперперфузии в компримированном отделе правой доли печени; б — изображение, реформатированное в косой сагиттальной плоскости, венозная фаза сканирования; образование широко прилежит, деформирует нижнюю полую вену (стрелки). Во время операции потребовалось выполнение пластики нижней полой вены вследствие инвазии в последнюю опухоли надпочечника.Figure 14. Computed tomography of patient M., 41 years old, with a large tumor of the right adrenal gland up to 90 mm in size: a — arterial phase of post-contrast scanning, diffusely uneven accumulation of the contrast agent, hyperperfusion zone in the compressed section of the right lobe of the liver; b — image reformatted in the oblique sagittal plane, venous phase of the scan; the mass is widely adjacent, deforming the inferior vena cava (arrows). During the operation, plastic surgery of the inferior vena cava was required due to invasion into the last tumor of the adrenal gland.

Необходимо отметить, что во всех анализируемых наблюдениях по совокупности изменений (плотность и размеры образования, наличие включений, характерные показатели контрастирования новообразования, инвазия опухоли), выявленных при КТ, позволили нам заподозрить АКР.

## ОБСУЖДЕНИЕ

## Репрезентативность выборок

Высокая репрезентативность выборок обуславливается тем, что набор участников исследования проводился только в федеральном научном центре, длительное время занимающемся лечением этой категории пациентов и имеющем единый стандартизированный протокол описания компьютерных томограмм.

## Сопоставление с другими публикациями

Клиническая значимость результатов

Нам не удалось установить ни одного фенотипического патогномоничного признака АКР при контрастном исследовании с внутривенным болюсным контрастированием. Вместе с тем КТ по стандартному протоколу должна быть проведена всем больным с подозрением или выявленным другим способом визуализации новообразованием надпочечников. Контрастное исследование с болюсным введением должно проводиться у всех пациентов в случае наличия денситометрической плотности выше +5 Ед HU при оценке нативных изображений. Отказ от контрастного исследования возможен только при наличии противопоказаний (аллергическая реакция, непереносимость, патология почек).

## Ограничения исследования

Во избежание систематических и случайных смещений результатов исследования данные КТ анализировались ограниченным числом высококвалифицированных специалистов по стандартизированному алгоритму. При оценке нативной плотности опухолей надпочечника избегали попадания в зоны, содержащие некрозы, кальцинаты и кровоизлияния.

Направления дальнейших исследований

В дальнейшем планируется изучение КТ-характеристик феохромоцитом в большой группе пациентов, проходивших обследование в одном медицинском центре.

## ЗАКЛЮЧЕНИЕ

Для АКР наиболее характерны: неоднородная структура опухоли, определяемая при 84,3% исследований; денситометрическая плотность опухолей, выявляемая при нативном сканировании, в интервале от +7,2 HU до +45 HU, при этом в ¾ наблюдений (75,5% случаев) нативная плотность была выше +30 HU; особенности вымывания контраста: показатель абсолютного контрастного вымывания менее 60% (68,6% наблюдений), показатель относительного контрастного вымывания менее 40% (64,6% случаев); размеры опухоли от 3 до 9 см — 75% наблюдений; признаки инвазии в окружающие анатомические структуры (10% случаев).

Первичная диагностика АКР происходит при средних размерах опухоли 7,05 (5,85–7,67) см. Большие размеры опухолей, выявляемые в настоящий момент (в половине наблюдений более 7 см, в четверти наблюдений (25%) более 9 см), свидетельствуют о выявлении заболевания на поздних стадиях, что существенно влияет на прогноз. Диагностика АКР у пациентов с размером опухоли менее 4 см встречается в 8,6% наблюдений, что имеет особое значение в клинической практике.

Несмотря на высокую информативность КТ как метода предоперационной диагностики АКР ­необходимо отметить, что все визуализирующие методы исследования косвенно оценивают физические, биохимические и метаболические свойства конкретной опухоли, и окончательное заключение о возможной ее морфологической структуре носит различной степени приближенности вероятностный характер. Поэтому использование исключительно данных КТ для дифференциальной диагностики этих новообразований не представляется возможным. В связи с чем предоперационная диагностика должна быть обязательно дополнена тщательным изучением клинической картины, данных лабораторных исследований и при необходимости должна дополняться позитронно-эмиссионным исследованием, которое, впрочем, также имеет ограничения.

## ДОПОЛНИТЕЛЬНАЯ ИНФОРМАЦИЯ

Источники финансирования. Исследование выполнено при финансовом обеспечении Санкт-Петербургского Государственного университета.

Конфликт интересов. Авторы декларируют отсутствие явных и потенциальных конфликтов интересов, связанных с содержанием настоящей статьи.

Участие авторов. Русаков В.Ф. критерий 1 — существенный вклад в концепцию исследования критерий 2 — написание статьи; Щербаков И.Е.: критерий 1 — существенный вклад в концепцию исследования, критерий 2 — написание статьи; Чинчук И.К.: критерий 1 — в получение, анализ данных или интерпретацию результатов, критерий 2 — внесение в рукопись существенной (важной) правки с целью повышения научной ценности статьи; Савельева Т.В.: критерий 1 — в получение, анализ данных или интерпретацию результатов, критерий 2 — внесение в рукопись существенной (важной) правки с целью повышения научной ценности статьи; Реброва Д.В.: критерий 1 — существенный вклад в концепцию исследования, критерий 2 — внесение в рукопись существенной (важной) правки с целью повышения научной ценности статьи; Смирнова О.И.: критерий 1 — в получение, анализ данных или интерпретацию результатов; критерий 2 — внесение в рукопись существенной (важной) правки с целью повышения научной ценности статьи; Придвижкина Т.С.: критерий 1 — в получение, анализ данных или интерпретацию результатов, критерий 2 — внесение в рукопись существенной (важной) правки с целью повышения научной ценности статьи; Черников Р.А.: критерий 1 — существенный вклад в концепцию исследования, критерий 2 — внесение в рукопись существенной (важной) правки с целью повышения научной ценности статьи; Краснов Л.М.: критерий 1 — существенный вклад в концепцию исследования, критерий 2 — внесение в рукопись существенной (важной) правки с целью повышения научной ценности статьи; Федотов Ю.Н.: критерий 1 — существенный вклад в концепцию исследования, критерий 2 — внесение в рукопись существенной (важной) правки с целью повышения научной ценности статьи; Федоров Е.А.: критерий 1 — в получение, анализ данных или интерпретацию результатов; критерий 2 — внесение в рукопись существенной (важной) правки с целью повышения научной ценности статьи; Федоров Е.А.: критерий 1 — в получение, анализ данных или интерпретацию результатов, критерий 2 — внесение в рукопись существенной (важной) правки с целью повышения научной ценности статьи; Саблин И.В.: критерий 1 — в получение, анализ данных или интерпретацию результатов, критерий 2 — внесение в рукопись существенной (важной) правки с целью повышения научной ценности статьи; Слепцов И.В.: критерий 1 — существенный вклад в концепцию исследования, критерий 2 — внесение в рукопись существенной (важной) правки с целью повышения научной ценности статьи; Шихмагомедов Ш.Ш.: критерий 1 — в получение, анализ данных или интерпретацию результатов, критерий 2 — внесение в рукопись существенной (важной) правки с целью повышения научной ценности статьи; Згода Е.А.: критерий 1 — в получение, анализ данных или интерпретацию результатов, критерий 2 — внесение в рукопись существенной (важной) правки с целью повышения научной ценности статьи.

Все авторы одобрили финальную версию статьи перед публикацией, выразили согласие нести ответственность за все аспекты работы, подразумевающую надлежащее изучение и решение вопросов, связанных с точностью или добросовестностью любой части работы

Благодарности. Благодарность Перфильевой Марии Сергеевне за помощь в составлении электронной базы данных.
